# Physicochemical composition of *Tamarindus indica* L. (Tamarind) in the agro‐ecological zones of Uganda

**DOI:** 10.1002/fsn3.627

**Published:** 2018-04-16

**Authors:** Jaspher Okello, John B. L. Okullo, Gerald Eilu, Philip Nyeko, Joseph Obua

**Affiliations:** ^1^ National Agricultural Research Organisation (NARO) Entebbe Uganda; ^2^ School of Forestry Environmental and Geographical Sciences College of Agricultural and Environmental Sciences Makerere University Kampala Uganda

**Keywords:** agro‐ecological zones, land use types, on‐farm, physicochemical composition, tamarind, *Tamarindus indica*

## Abstract

The relationships between the physicochemical composition of *Tamarindus indica* pulp and seeds, and agro‐ecological zones and land use types were assessed in Uganda. The objective was to determine the relationship between the physicochemical composition, agro‐ecological zones, and land use types. The samples were processed by manually depulping the *T. indica* pods, sun‐drying the pulp and seeds, and grinding into powder. The powdered samples were analyzed for β‐carotenoids, vitamin C (ascorbic acid), calorific value, crude oil, acid, and peroxide values. Data were analyzed using ANOVA in the general linear model (GLM). Principal component analysis (PCA) was used to relate the physicochemical properties to the agro‐ecological zones and land use types. There were significant differences (*p* ≤ .05) in the physicochemical composition variables between agro‐ecological zones and land use types. Land use types showed strong correlations with physicochemical properties while agro‐ecological zones did not show correlations. The results show that in terms of general properties, *T. indica* pods provide a valuable, rich, and exceptional source of vitamin C, compared to many widely consumed indigenous and conventional fruits and vegetables. The pods from land use types characterized by natural habitats had relatively more nutrient levels than the land use types influenced by anthropogenic activities.

## 1 INTRODUCTION

*Tamarindus indiica* LINN (syn. *T. occidentalis* Gaertn.; *T. officinalis* Hook.) of Fabaceae family, subfamily Caesalpinioideae, is a tree species belonging to the monotypic genus, Tamarindus. The common English name is “Tamarind.” It is an important multipurpose indigenous fruit tree that is indigenous to Uganda and Africa. It is valued mainly for the pods that are eaten fresh or processed for various uses. Other parts of the fruit are used, because they contain minerals and nutrients (Okello, Okullo, Eilu, Nyeko, & Obua, 2017). The fruits contain about 30% pulp, 40% seeds, and 30% hull (Singh, Wangchu, & Moond, 2007). The pulp is rich in sugar (30%–40%) compared to many other conventional and indigenous fruits. Due to its relatively high sugar concentration and low pH, the pulp can be used industrially as concentrates, pickles, confections, and powders (Sulieman, Alawad, Osman, & Abdelmageed, 2015). It is used to treat meningitis and other ailments in the West Nile and other parts of Uganda. The tree species is also considered a perfect agroforestry tree due to its deliberate retention and presence on‐farm, association with both annual and perennial crops and also grows wild across all the agro‐ecological zones of Uganda (Okello et al., 2017) as well as high‐altitude areas above 2,000 masl such as mountain ranges.

Many studies have determined the physicochemical composition of common fruits and vegetables (Ajayi, 2010; MFAF, 2014; Singh et al., 2007; Tagoe, Dickinson, & Apetorgbor, 2012). However, in Uganda, *T. indica*, an indigenous species has been neglected, indiscriminately grows without much attention at anywhere like on‐farm, wild, roadsides, abandoned homesteads, market places but are cheap and are commonly consumed by rural population. The use of *T. indica* pulp, leaves, and flowers for food, medicine, and other industrial purposes (e.g., juice, concentrate, powder, pickles, and paste) has been reported by Singh et al. (2007). Okello et al. (2017) reported that the *T. indica* fruits provide two important products—pulp, mostly eaten directly or used for making local food, drinks, and sold for domestic incomes, whereas the seeds, obtained after depulping the pod, are usually thrown away due to total lack of knowledge on its properties and uses.

Whereas there is information on the physicochemical properties of many neglected indigenous tree species (Abiodun, Dauda, Adebisi, & Alonge, 2017; Ajayi, 2010; Hiremath, Yadav, & Suguna, 2016; Ouilly et al., 2017; Sulieman et al., 2015), many studies focused on the edible parts (pod pulp and leaves). Little attention was given to non‐edible parts such as seeds. Furthermore, the relationship between the physicochemical characteristics and growth conditions within the different agro‐ecological zones and land use types has not been investigated despite reported uses. Thus, the objective of the present study was to determine the *T. indica* physicochemical composition of *T. indica* in relation to agro‐ecological zones and land use types in Uganda. It was hypothesized that there were no significant differences in the physicochemical characteristics of *T. indica* pulp and seeds between agro‐ecological zones and land use types. The findings of this study are useful for guiding the utilization and land use‐related decisions.

## 2 MATERIALS AND METHODS

### 2.1 Agro‐ecological zones of Uganda and study sites

Uganda, officially the Republic of Uganda, is located astride the Equator in Eastern Africa covering 2,41,550.7 km^2^. It lies between latitude 4^°^12′N and 1^°^29′S and longitude 29^°^34′E and 35^°^0′E. Most of Uganda lies within the interior plateau of the Africa continent. The predominant rocks were formed between 3,000 and 6,000 million years ago (pre‐Cambrian era). However, in some western and eastern parts of the country, there are major developments of younger rocks, which are either sediments or volcanic in origin ranging from 135 million years ago (Cretaceous period) to the present day (NEMA, 2007). The types and characteristics of the soils of Uganda are defined by parameters such as nature of the parent rock, age of the form, and climate (especially the amount of moisture). The ferralitic soils (ferralsols) are the most dominant. Others are ferruginous, volcanic, and alluvial soils (NEMA, 2007). The climate is tropical, with distinct wet and dry seasons. In many parts of the country, the dry season occurs between December and March.

**Figure 1:**
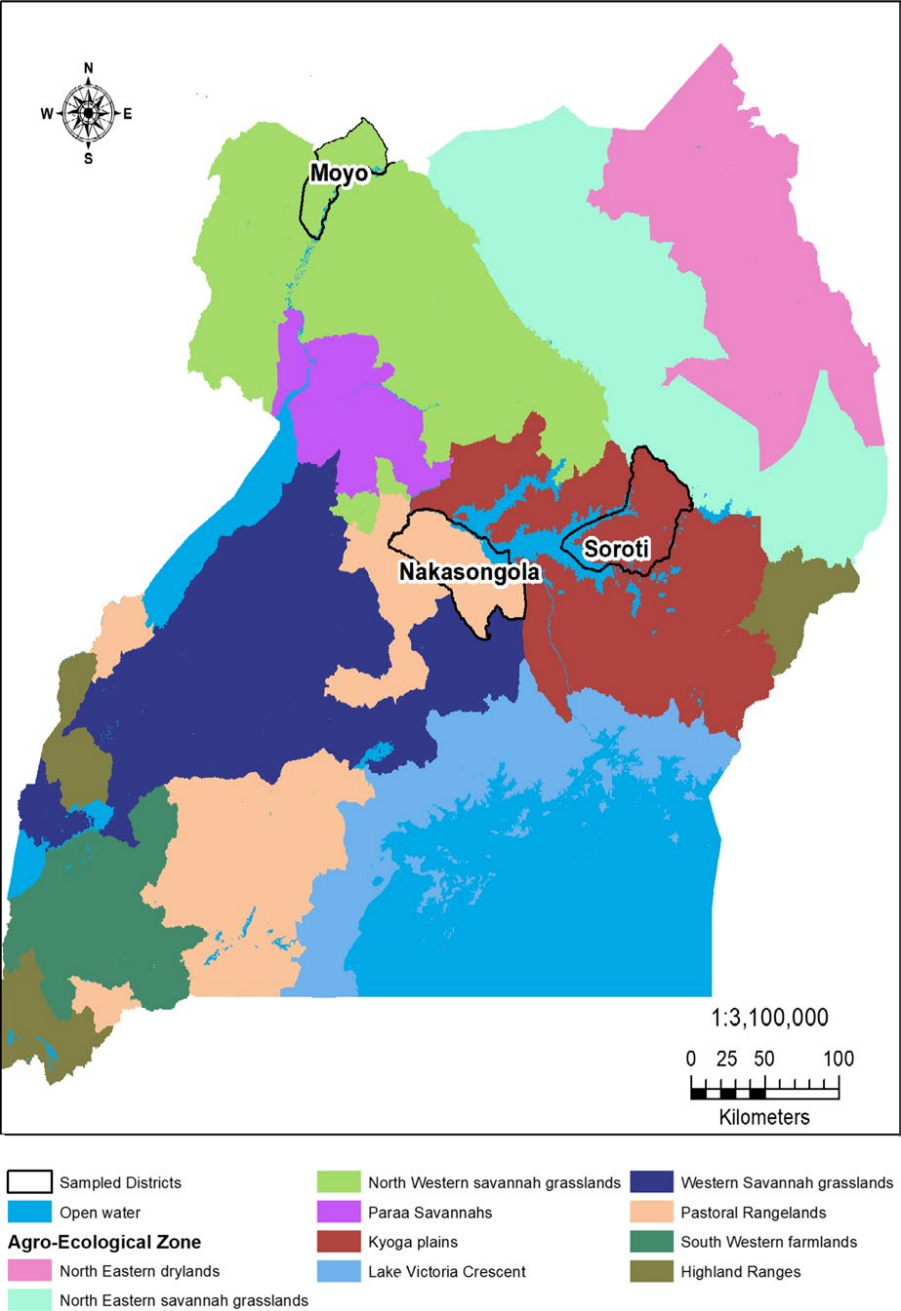
Map of Uganda showing agro‐ecological zones and sample sites

Uganda is divided into nine agro‐ecological zones primarily based on agro‐climatic factors (rainfall totals and distribution) and soils (productivity and fertility). Topography, temperature, moisture, and vegetation cover are the secondary factors characterizing a zone but differing between zones (GoU, 2004). Hence, the climate, geological formation, topography, soil types, rainfall, and farming systems or practices are fairly homogeneous within zones. This study was conducted in the three agro‐ecological zones (Eastern, West Nile, and Lake Victoria Crescent) where *T. indica* trees occur naturally on‐farm and in the wild. Each zone was represented by a district, that is, Soroti, Moyo, and Nakasongola for Eastern, West Nile, and Lake Victoria Crescent agro‐ecological zones, respectively (Figure 1). Uganda is divided into 121 districts (including the capital city of Kampala), which are grouped into four administrative regions. The district is the largest decentralized unit of administration in the country.

Nakasongola district is located in Central Uganda in the Lake Victoria Crescent agro‐ecological zone. It covers 3,510 km^2^, and the altitude ranges between 1,000 and 1,400 masl. The topography is generally flat, characterized by small altitudinal differences with poor drainage in the wide flat valleys and Lake shores. It is endowed with unique rocky outcrops (isenbergs). The soils are mostly shallow and skeletal having developed from quartzite or iron stone (ferralitic). The vegetation type is open deciduous savanna woodland. There are two rainy seasons (March–July and August–November), with bimodal total rainfall of between 875 and 1,000 mm per annum. Minimum and maximum temperatures are 18 and 28^°^C, respectively (NEMA, 2004a).

Soroti district is located in Eastern Uganda in the Eastern agro‐ecological zone, covering an area of 2,662.5 km^2^ and altitude of 1,036–1,127 masl. The major rocks are granites, magnalites, gneiss, schists, and quartzites. The district is mainly underlain by rocks of the basement complex pre‐Cambrian age. The major soils are of the Serere and Amuria catena, Metu complex, and Usuk series with moderate agricultural productivity. The vegetation types include wooded savanna, grassland savanna, forest, and riparian. Two rainy seasons occur from March to June and August to November with total annual rainfall of 1,000–1,500 mm, with December and January being the driest months. The minimum and maximum temperatures are 18 and 31.3^°^C, respectively (GoU, 2014).

Moyo district is located in the West Nile region of Uganda within the West Nile agro‐ecological zone. It covers an area of 1,891 km^2^ with an altitudinal range of 600–1,586 masl. The topography is characterized by low plains as well as rolling hills and valleys that slope toward River Nile. A series of hills and peaks characterize the northern and northeastern parts of the district (NEMA, 2004b). The major geological formations are the gneiss, alluvial deposits, schists, quartzite, and marble that occur in the mountains. The major soil types include the vertisols, leptosols, alluvial deposits, and ferralsols that are moderately fertile (NEMA, 2004b). The rainfall varies between 1,500 and 1,700 mm, less pronounced, bimodal, and occurring mainly in March to June and August to November with the dry season in late November to early March. The minimum and maximum temperatures (23.7–30^°^C) are of modified equatorial type (UDIH, 2007). The vegetation is classified as wooded savanna.

### 2.2 Methods

#### 2.2.1 Sampling design

The sampling sites were located within an ecological gradient that took account of differences in climate and agro‐ecology. The three sample agro‐ecological zones (Eastern, West Nile, and Lake Victoria Crescent) are located more than 300 km apart. Each agro‐ecological zone was stratified into two major land use types: crop fields (on‐farm) and wild lands. The crop fields (on‐farm) are farmlands with agricultural crops, while the wild land had not been cultivated for 5 or more years prior to the study.

Four representative sub‐counties (sample sites) were selected in each land use type in each district making a total of twelve study sites (four study sites per district) covering about 5 km^2^. Up to five *T. indica* sample trees were randomly selected per land use type in each sampling site based on ease of access, absence of obvious signs of pests, diseases and fire, and presence of good mature pods. The sample trees were located at least 200 meters apart to avoid sampling siblings.

#### 2.2.2 Sample collection methods

The pods were collected during the dry season between December and March. Fruits were collected from the top, middle, and bottom parts of the tree canopy. The trees were climbed using ladders. Ripe pods were selected by gently squeezing them. The ripe pods had scurfy brown, woody, fragile shells with brown pulp that cracked on squeezing. The stalk of the ripe pod was severed with a knife to remove the pod without causing damage to the pod, developing flowers and leaves.

Eight pods were collected from each canopy level making 24 pods from each tree. A total of 480 pods were harvested from 20 trees per land use type, giving 960 pods per district (zone) and 2,880 pods from the three agro‐ecological zones. Pods collected from each tree were pooled, kept in white polythene bags and labeled. The samples were then taken to Makerere University's College of Agricultural and Environmental Sciences laboratory for physicochemical analyses.

#### 2.2.3 Samples preparation for laboratory analyses

The pods were washed with distilled water and allowed to dry for 1 hr to maintain constant moisture content of the shell. Pod shells were manually depulped, and morphological traits such as pod length, pod breadth, pod total mass, pod seed number, total seed mass, and pulp mass were determined. All measured samples were pooled by land use types and agro‐ecological zones for proximate analyses.

Decomposed and damaged pulp and seeds were discarded. The depulped seeds and pulp were separately sun‐dried for 6 hr/day for 3 days to lower the moisture content and later dried in an oven at 40°C for 3 days to 8% moisture content. These were then separately grounded in an electric grinding machine (Brooks Crompton, 2000 series—UK) to 60‐mesh size. The powdered samples were stored in ziplock plastic bags at room temperature for further laboratory analyses of β‐carotenoids, vitamin C (ascorbic acid), crude oil, acid (gmg^‐1^), and peroxide (mEq/kg), whose values are very important for human and animal nutrition.

### 2.3 Crude oil extraction and physicochemical analyses

The powder from pulp and seeds was separately analyzed for β‐carotenoids and vitamin C (ascorbic acid). The β‐carotenoids were analyzed under subdued light following Rodriguez‐Amaya and Kimura (2004), while vitamin C was determined by titration based on AOAC (1999). Oil was first extracted using the soxhlet methods (AOAC, 1999) and then subjected to physical and chemical characterization. The acid value (gmg^‐1^) and peroxide value (mEq/kg) were determined using the analysis of rancidity (Kirk & Sawyer, 1989). All physicochemical composition analyses were performed in triplicate and the average reading recorded.

### 2.4 Data analysis

**Figure 2:**
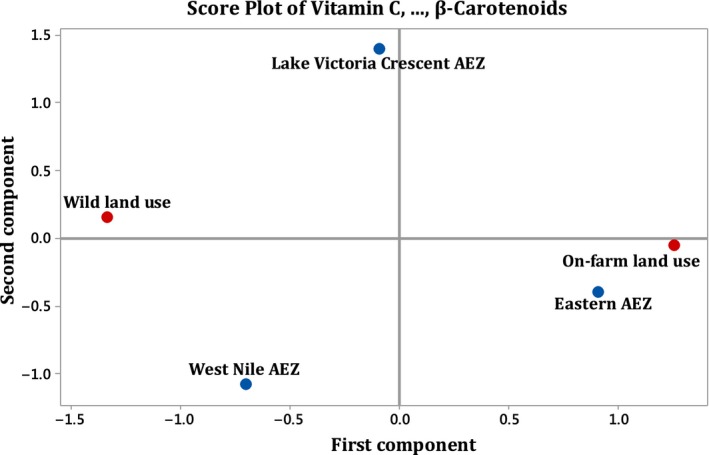
Principal component analysis for *T. indica* pulp samples

**Figure 3:**
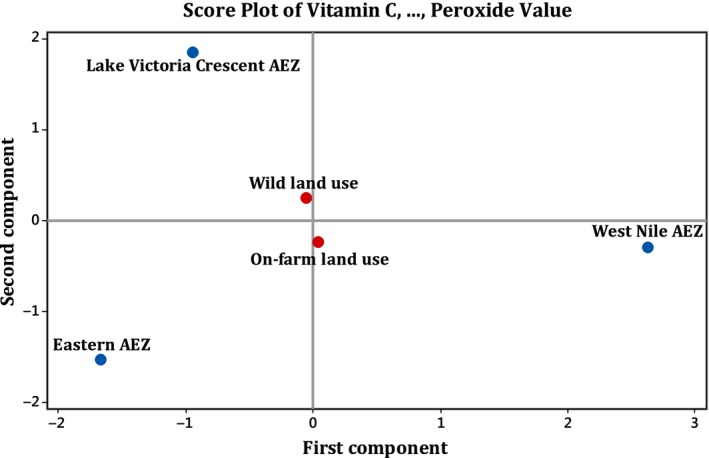
Principal component analysis for *T. indica* seed samples

Data were entered into Microsoft Excel by agro‐ecological zone, land use type and source of samples (pulp and seed). Univariate analyses of variance (ANOVA) in the General Linear Model (GLM) were carried out in SPSS for windows version 16.0 to determine the relationship between physicochemical composition, agro‐ecological zones and land use types. Treatment means were separated using the Least Significant Difference (LSD) in Post Hoc Tests at the probability level of 5%. Mean data of triplicate analyses and standard deviations are presented in Tables 1 and 2. The Principal Component Analyses (PCA) were carried out to relate the physicochemical properties to the agro‐ecological zones and land use types by reducing the numerous dimensions into a few axes that could be interpreted with certainty. The PCA analyse and outputs are presented in Tables 3 and 4 and ordination diagrams (Figures 2 and 3). Variables that had loadings of 0.50 or greater were considered to be making, at minimum, a reasonable contribution to the principal component. In addition, the second principal component is independent of the first principal component and the third is independent of the first two principal components, and so on.

**Table 1:** Physicochemical composition of *Tamarindus indica* pulp from different agro‐ecological zones and land use types in Uganda

Nutrients	Agro‐ecological zones (mg/100 g)			Land use types		Agro‐ecological zones × Land use types						*p* Value	SE Value
	West Nile AEZ	LVC AEZ	Eastern AEZ	Wild	On‐farm	LVC* Wild	LVC*On‐farm	East *Wild	East*On‐farm	WN*Wild	WN*On‐farm	AEZ* Land Use	AEZ* Land Use
Vitamin C	201.70 ± 0.02^a^	138.40 ± 0.01^b^	154.60 ± 0.03^c^	189.40 ± 0.35^a^	140.40 ± 0.21^b^	140.50	136.30	157.80	151.40	270.00	133.30	.001	4.514
β‐Carotenoids	0.16 ± 0.02^a^	0.14 ± 0.02^b^	0.17 ± 0.03^c^	0.14 ± 0.03^a^	0.17 ± 0.04^b^	0.15	0.12	0.16	0.18	0.11	0.21	.001	0.001

The contents are expressed by the mean values ± *SD* for *n* = 3 and *n *=* *2. AEZ, Agro‐ecological zone.

*Interactions between land use types and agro‐ecological zones with variables; same superscript letters within a row show no significant difference.

**Table 2:** Physicochemical composition of *Tamarindus indica* seed from different agro‐ecological zones and land use types in Uganda

Nutrients	Agro‐ecological zone (Contents/100 g)			Land use types		Agro‐ecological zones × Land use types						*p* Value	SE Value
	West Nile AEZ	Lake Victoria Crescent AEZ physicochemical	Eastern AEZ	Wild	On‐farm	LVC* Wild	LVC*On‐farm	East* Wild	East*On‐farm	WN* Wild	WN*On‐farm	AEZ* Land Use	AEZ* Land Use
Vitamin C	103.20 ± 0.14^a^	85.10 ± 0.70^b^	104.80 ± 0.57^a^	106.20 ± 1.00^a^	89.20 ± 0.70^b^	88.10	82.00	132.00	77.80	98.50	107.80	.001	4.484
β‐carotenoids	0.13 ± 0.20^a^	0.24 ± 0.12^b^	0.33 ± 0.13^c^	0.20 ± 0.02^a^	0.27 ± 0.02^b^	0.16	0.32	0.32	0.34	0.13	0.13	.001	0.001
Calorific value	280.90 ± 1.07^a^	265.00 ± 1.00^b^	267.80 ± 2.05^c^	269.00 ± 1.53^a^	273.40 ± 0.84^b^	257.70	272.30	272.00	263.40	277.20	284.60	.001	0.026
Acid value	10.00 ± 1.50^a^	19.30 ± 0.40^b^	19.50 ± 0.31^c^	18.80 ± 0.60^a^	13.70 ± 0.36^b^	23.00	15.50	25.10	13.80	8.40	11.70	.001	0.029
Peroxide value	200.30 ± 0.80^a^	235.10 ± 1.00^b^	111.00 ± 0.53^c^	208.80 ± 1.22^a^	155.50 ± 1.04^a^	353.80	116.40	148.00	74.10	124.80	275.90	.001	0.008

The contents are expressed by the mean values ± *SD* for *n* = 3 and *n* = 2. AEZ, Agro‐ecological zone.

*Interactions between land use types and agro‐ecological zones with variables; same superscript letters within a row show no significant difference.

**Table 3:** The PCA Eigenvectors for *T. indica* Pulp

Variable	Principal component	
	PC1	PC2
Vitamin C	**−0.707**	**−0.707**
β‐carotenoids	**0.707**	**−0.707**

Bold letters signifies strong correlations.

**Table 4:** The PCA Eigenvectors for *T. indica* Seed

Variable	Principal Component				
	PC1	PC2	PC3	PC4	PC5
Vitamin C	0.134	**−0.542**	**0.772**	**−**0.298	0.070
β‐carotenoids	**−0.549**	**−**0.273	**−**0.327	**−0.664**	**−**0.276
Calorific value	**0.568**	**−**0.274	**−**0.202	0.053	**−0.747**
Acid value	**−0.557**	0.147	0.416	0.428	**−0.559**
Peroxide value	0.219	**0.732**	0.290	**−0.533**	**−**0.219

Bold letters signifies strong correlations. Correlations above 0.5 is deemed important.

## 3 RESULTS

### 3.1 Physicochemical composition of *T. indica* pulp samples

Generally, there were differences between agro‐ecological zones and between land use types. The physicochemical composition values of the pulp were higher in the West Nile agro‐ecological zone samples as well as the samples collected from the wild land use types. Specifically, there were significant differences (*p* ≤ .05) between agro‐ecological zones and between land use types for β‐carotenoids as well as vitamin C. The samples from the West Nile zone had significantly higher vitamin C contents compared to samples from other agro‐ecological zones. However, β‐carotenoids were nearly similar in all the agro‐ecological zone samples (Table 1).

### 3.2 Physicochemical composition of *T. indica* seed samples

The physicochemical composition of *T. indica* seed samples showed significant differences (*p* ≤ .05) between agro‐ecological zones. All properties were significantly different between land use types with the exception of peroxide value. The absolute amounts of different components were nearly similar in all agro‐ecological zones and land use types (Table 2).

### 3.3 Relating the physicochemical properties of pulp samples to agro‐ecological zones and land use types

The first principle component (PC1) is strongly correlated with all the two original variables (β‐carotenoids and vitamin C). It also shows a perfect contrast between these variables (β‐carotenoids and vitamin C). The first principal component increases with increasing β‐carotenoids and decreasing vitamin C contents (Table 3). Based on the correlations of +0.707 and −0.707, this component is primarily a measure of the quality of β‐carotenoids and poor contents of vitamin C respectively. Both the β‐carotenoids and vitamin C have greater impacts on the component. This shows that *T. indica* samples with high β‐carotenoids values have high quality β‐carotenoids contents in terms of α‐ and β‐carotenes, which are precursors of vitamin A**.** Accordingly, this component can also be viewed as a measure of how poor contents of vitamin C affects its functions in humans (prevention and treatment of scurvy, an antioxidant and co‐factor functions for enzyme metabolism), and plant (co‐factor for enzymes involved in photosynthesis and synthesis of plant hormones).

In the second principal component (PC2), strong correlations with both vitamin C and β‐carotenoids (−0.707) were observed. This second component is primarily a measure of vitamin C and β‐carotenoids based on the strong negative correlations. The second component increases with decreasing vitamin C and β‐carotenoids. These two variables (β‐carotenoids and vitamin C) have greater impacts to the component. This component can be viewed as a measure of the poor quality of β‐carotenoids and poor contents of vitamin C to adequately alleviate the health of humans and the roles it plays in plant functioning. The PCA ordination result shows that only West Nile agro‐ecological zone strongly influences the component and weak influences/relations were observed for land use types and physicochemical properties. In addition, on‐farm land use showed weaker influence than wild land use on the component, all agro‐ecological zones are further apart, quite dissimilar and did not show any close to average values but instead showed multivariate outliers (extreme observations). However, only on‐farm land use type and Eastern agro‐ecological zone having similar observations (Figure 2).

### 3.4 Relating the physicochemical properties of seed samples to agro‐ecological zones and land use types

The first principal component (PC1) is strongly correlated with three of the original variables (β‐carotenoids, calorific and acid values). The component also has large positive and negative associations with these three variables as shown in Table 4. These three original variables have more roles to play in the component than other variables. The first principal component increases with increasing calorific values and decreasing β‐carotenoids and acid values. These suggest that these three criteria vary together – if one increases, then the remaining ones tend to increase as well. Thus, based on the strong correlations of 0.568, −0.557 and −0.549, this component is a measure of calorific values (quality of energy food provided by water, proteins, fats and carbohydrates), acid values (how unhealthy poor quality oil is) and β‐carotenoids contents (poor quality of α‐ and β‐carotenes, which are precursors of vitamin A). Unlike other variables, vitamin C and peroxide values have less roles to play in the component.

The second principal component (PC2) is strongly correlated with vitamin C and peroxide values. This component increases with only two of the original variables, increasing peroxide values and decreasing vitamin C contents. β‐carotenoids, calorific values and acid values have less roles to play in this component. Based on the strong correlation of 0.732, it can be stated that PC2 is a measure of low oxidation of oils (peroxide values). While PC3 increases with only one original variables, increasing vitamin C contents, the rest of the variables have less roles to play in the component. The strong correlation of 0.772 is an indication that PC3 is primarily a measure of high contents of vitamin C in the samples.

The fourth component is strongly correlated with only two of the original variables (β‐carotenoids and peroxide values). The component increases with decreasing β‐carotenoids and peroxide values. Due to its strong correlation of −0.664, this principal component is primarily a measure of poor quality α‐ and β‐carotenes, which are precursors of vitamin A. This also suggests that β‐carotenoids and peroxide values have more roles to play in the component than other original variables. On the other hand, PC5 is strongly correlated with calorific and acid values, where the component is increasing with decreasing calorific and acid values. This component is a measure of calorific and acid values which have more roles to play in the component than any other variables. While PC1 accounts for 8.44% of the overall variability, PC2 explains 9.58% of it and PC3, PC4 and PC5 explains 43.31%, 46.28% and 79.00% overall variabilities respectively. In general, the PCA analysis of *T. indica* seed samples revealed weak correlations with land use types and the further apart of all agro‐ecological zones is not a show of similarity but outliers/extreme observations (Figure 3).

## 4 DISCUSSION

### 4.1 Vitamin C (ascorbic acid)

The amount of vitamin C (ascorbic acid) recorded was 138.4–201.7 mg/100 g and 85.1–104.8 mg/100 g in the *T. indica* pulp and seed samples, respectively. In general, the pulp samples exhibited higher vitamin C contents (about twice) compared to the seed samples. Vitamin C is water‐soluble and cannot be stored by the body except in insignificant amounts and is obtained mainly through the consumption of fresh fruits and vegetable (SLH, 2017). The human body requires it for the prevention and treatment of scurvy, an antioxidant, and cofactor functions for enzyme metabolism while plants use it as cofactor for enzymes involved in photosynthesis and synthesis of plant hormones. The values recorded in this study were higher than those reported by Sadiq, Duruminiya, Balogun, Kwada, and Izuagie (2016); Sulieman et al. (2015); Chiteva and Kituyi (2006) and El‐Siddig et al. (2006). Vitamin C contents of other documented plant species were within the range reported for *Dialium guineense* (Abiodun et al., 2017) but lower than values reported for olive fruit (Muhammad et al., 2014).

High vitamin C contents recorded in the samples especially pulp probably show the freshness of the samples, which corroborates the report of SLH (2017) that indicated high contents in fresh fruits and vegetables. According to Dimari and Hati (2010), a number of researchers have attributed factors such as fruit type and storage methods, temperature, microorganism, and package type on the stability of vitamin C. Hiremath et al. (2016) recorded a reduction in vitamin C content after processing in comparison with the fresh counterparts. The Natural Food Hub (2017) shows the kiwifruit tree species as an exceptionally rich source of vitamin C but a kiwifruit that has been cool stored for a while has reduced vitamin C content. Additionally, Liji (2016) reported that the availability of vitamin C is reduced by cooking and long periods of storage.

The differences observed in terms of the contents of vitamin C in samples analyzed are probably as a result of the plant parts used, position of fruits on the tree, storage conditions, stages of fruit maturity and ripeness, climate factors, effects of environmental conditions, length of time since samples were picked, methods of preparations and plant management conditions (on‐farm and wild). This agrees with Okello et al. (2017) who documented different growing climatic and environmental factors, plant parts used, plant management conditions, and length of storage before analysis. Additionally, Rasanu, Magearu, Matei, and Soceanu (2005) reported that the amount of vitamin C in fruit depends on the precise variety of the fruit, the soil and climate in which it grew, and the length of time since it was picked. While Mahdavi, Nikniaz, Rafraf, and Jouyban (2010) documented differences in production factors and climatic conditions, maturity state and position on the tree, type of fruits (species and variety), handling and storage. In citrus study, Muhammad et al. (2014) reported the position of citrus fruits on the tree affects vitamin C levels.

According to Muhammad et al. (2014), vitamin C levels in the unripe fruits are higher than the ripe ones but generally decreased upon increase in temperature, ripening, and time of exposure. While species such as *Ziziphus jujube* fruit, the vitamin C content does the opposite—it rises with increased ripeness (The Natural Food Hub, 2017). Kaleem et al. (2016) also reported that at increased temperature, the amount of vitamin C decreases, due to oxygen, the most destructive element in fruit juice causing degradation of vitamin C in fruits. At increased temperature, the juice is more susceptible to oxidation and the effects of temperature and consequently experience more degradation of vitamin C. The vitamin C decreases upon ripening, temperature increase, and time which are attributed to degradation caused by heat and oxidation. Also, as the temperature increases, the vitamin C content decreases. The minimum daily requirement of vitamin C for preventing clinical symptoms of the specific deficiency‐scurvy for adults is 45 mg/100 g. The contents of vitamin C in our study are well above the recommended dietary allowance (RDA) values, and the daily intake of 100 gram pulp or seed can prevent scurvy and other vitamin C‐related ailments in Uganda. Additionally, Uganda's *T. indica* are valuable, rich, and exceptional source of vitamin C than most indigenous and conventional fruits and vegetables and can contribute substantial amounts of vitamin C toward the consumers’ dietary needs.

### 4.2 Beta carotenoids (β‐carotenoids)

The amount of β‐carotenoids in *T. indica* pulp and seed samples were 0.14–0.17 mg/100 g and 0.13–0.33 mg/100 g, respectively, in this study. The most important of these are α‐ and β‐carotene, which are precursors of vitamin A. Of the carotenes, β‐carotenoid has the strongest provitamin A activity. Carotenoids are important in human health. Vitamin A deficiency can lead to blindness, and the use of *T. indica* pulp and seed samples is good prophylactic of this condition. β‐carotenoids is the main safe dietary source of vitamin A, essential for normal growth and development, immune system function, healthy skin and epithelia and vision (Institute of Medicine, Food and Nutrition Board, 2000). The β‐carotenoid has antioxidant properties that can help neutralize free radicals—reactive oxygen molecules potentially damaging lipid in cell membranes, proteins, and DNA. In this study, we reported lower values than values reported for *Lannea kerstingii* (Ouilly et al., 2017), *Dialium guineense* (Abiodun et al., 2017), and tropical leafy vegetables (Djuikwo, Ejoh, Gouado, Mbofung, & Tanumihardjo, 2011). De Caluwe, Halamova, and Van Damme (2010) reported low carotenoids in *T. indica* fruit pulp.

The UMMC (2017) reported the rich sources of β‐carotenoids are yellow, orange, and green leafy fruits and vegetables (such as carrots, spinach, lettuce, tomatoes, sweet potatoes, and broccoli). In fruits, carotenoids increase markedly both in numbers and in quantity during ripening. The more intense the color of the fruit or vegetable, the more the β‐carotenoid it has. The pulp and seeds in this study are not rich in β‐carotenoids, attributed partly to their non‐green colouration, effects of land use practices (cultivated and wild species), stage of maturity of pods, climatic and environmental factors of the growing areas, harvest and postharvest handling processes, processing and storage conditions. Hiremath et al. (2016) reported the reduction in β‐carotene content after processing in comparison with the fresh counterparts. Although household preparation and processing procedures, such as boiling and drying, can help liberate the β‐carotenoids from their cellular matrix and enabling them to be available for absorption, these processes can lead to significant losses for β‐carotenoids in processed tropical leafy vegetables during sun‐drying (Djuikwo et al., 2011). However, the composition of β‐carotenoids in fruits and vegetables is more complex and variable, with variations in the principal carotene (Rodriguez‐Amaya & Kimura, 2004). While there is a RDA for vitamin A, there is no RDA for β‐carotene. Our study results show that the species could be used as a source of provitamin A to prevent night blindness in children.

### 4.3 Acid value

Acid and peroxide indexes are parameters that demonstrate the quality of the oil (Muniz et al., 2015). Acid value of 10.0–19.5/gmg was recorded for this study. Akubugwo, Chinyere, and Ugbogu (2008) reported that the acid value indicates edibility of oil and its suitability for use in the paint industry. Fats and oils are graded by their acid and free fatty acid contents, which are used as an index to determine their quality (Kardash & Tur'yan, 2005). The values of the present study are higher than those documented for *Lannea kerstingii* (Ouilly et al., 2017) and *Allanblackia floribunda* (Wilfred, Adubofuor, & Oldham, 2010). However, our documented values are within the range reported by Taha, Nour, and Elkhalifa (2016) but lower than those values reported in other studies such as Ajayi (2010) and El‐Siddig et al. (2006). However, a study by Muhammad, Ayub, and Zeb (2013) documented low acid values for olive fruit (*Olea europaea*), while Kittigowittana, Wongsakul, Krisdaphong, Jimtaisong, and Saewan (2013) reported higher values for *Hervea brasiliensis*.

Oils processed from fresh fruits have low free fatty acids (low acid values) compared with those from many days’ old fruits. The antioxidant activities of the seed oils may be attributed to fatty acid components (Kittigowittana et al., 2013). Fatty acids, which are the major constituents of oils—may include saturated, monounsaturated, and polyunsaturated fatty acids which contribute to human physiology in different ways (Costa, Ballus, Teixeira, & Godoy, 2011; ). Fatty acids are part of the composition of membranes of all plant tissues and of reserve lipids. Most fatty acids are involved in the maintenance of membrane structures and thus in the survival of the plant at low temperature (Škorič, Sakač, & Demurin, 2015). According to Godswill et al. (2016), saturated fatty acids have specific fluidity characteristics: they serve in the composition of several dietary and non‐dietary fats like margarine. Vegetable oils with high saturated fatty acids’ contents are of great industrial interest because they limit the steps involving hydrogenation to obtain a pure source of saturated fatty acids. However, high amounts of saturated fatty acids at the level of all tissues of the entire plant are lethal, and as such, an increase in saturated fatty acid content is specific in seeds and fruits. Additionally, highly unsaturated vegetable oils are less suitable for many food applications (Satchithanandam et al., 2004).

A high proportion of the variation in fatty acids content is due to environmental factors (Lanna, José, Oliveira, Barros, & Moreira, 2005). Our study reported higher acid values in West Nile agro‐ecological zone with maximum temperature above 30°C than Lake Victoria Crescent agro‐ecological zone with maximum temperature below 30°C. All fats and oils in nature are a mixture of saturated, monounsaturated and polyunsaturated fatty acids ‐ the difference in them is the proportion of each. Whether in plant or animal tissues, fats and oils operate at different temperatures, the most important consideration which is often neglected when discussing their healthiness. The degree of saturation or unsaturation determines not only a fat's melting point, but also its chemical stability and its likelihood of auto‐oxidizing and creating harmful free radicals. The degree of saturation of plant oils and fats is entirely dependent on the temperature in which they are grown. The higher the proportion of saturated fatty acids a fat is, the less likely it is to go rancid; the more polyunsaturated fatty acids it contains, the more difficult it is to stop it going bad (Barry, 2011).

Fruits that have been stored for long periods prior to processing and determination have high free fatty acid contents. Fatty acids play a very important role in fats and oils because of their health implications in the human diet and properties in industrial processes. In addition, Ekop, Etuk, and Eddy (2007) reported an increase in the acid value of oil is an indication of the onset of rancidity. However, hydrogenated fats and oils prevent rancidity and are used in foods to improve texture and stability for a longer shelf life because *trans* fatty acids have higher melting points and greater stability than their *cis* isomers (Dixit & Das, 2012). And rancidity can be accelerated by moisture, air, and presence of some metals, which agrees with a report by Okello et al. (2017; 2010) who documented high levels of moisture content and mineral composition in the Lake Victoria Crescent agro‐ecological zone, which recorded high acid value.

Oil extraction and acid value determinations for our study were delayed, and this may explain the relatively high acid values recorded. Our study findings corroborate the findings of Tagoe et al. (2012) showing that free fatty acid content of tamarind oil was affected by the length of sample storage and the length of storage of the oil after processing. According to Tagoe et al. (2012), the type of fatty acid determines the nutritional status and storability (keeping quality) of the oil. Free fatty acid and acid value of the oil extract from *T. indica* seeds are high (high acid values) and could not be stored for a long time without spoilage through oxidative rancidity and could not find application as edible oils. Thus, oil extracts from the *T. indica* samples will require refining to make them edible. It is thus important to consider improving fatty acid composition of dietary *T. indica* oils to enhance health and safety.

### 4.4 Peroxide value

The peroxide values of *T. indica* seed oil samples ranges from 111.0 to 235.1 mEq/kg. Peroxide value indicates the degree of oxidation of the oil—low peroxide value indicates low oxidation (Ekop et al., 2007). The present study values are above the values reported for most common plants (Ajayi, 2010; Ekop et al., 2007; Muhammad et al., 2013; Ouilly et al., 2017; Wilfred et al., 2010). This value is above the range of peroxide values (<10 mEq/kg) generally reported for fresh fats and oils (Kirk & Sawyer, 1991). The value also falls above the standards (peroxide value <10 mEq/kg fat or oil) stated by Codex Alimentarius for edible oils. In addition, our study result's high values are in agreement with Muniz et al. (2015) for *Bertholletia excelsa* who documented the high acid value due to variations in the conditions of the oil conservation, such as changes in storage time, time for performing the assessments, as well as particularities during the extraction process, which resulted in an increased oxidation of the oil.

High values of peroxide exhibited by the samples could probably be due to increased oxidation rate, associated with delayed *T. indica* oil extraction and later delayed determination of peroxide values. The high peroxide values are indicative of high levels of oxidative rancidity of the oils and suggest absence or low levels of antioxidant. These results corroborate with Kyari (2008) who reported that an increase in the corresponding peroxide values is much greater for the oil which was stored in the light, suggesting a high level of photo‐catalyzed oxidation of the oil. According to FAO (1982), the permitted peroxide values of not more than 10 mEq/kg have been reported for soybean, cotton seed, rapeseed, and coconut oils. In addition, the presence of antioxidants (such as vitamin C) in the plant parts analyzed have effects on the observed peroxide values. This shows that the oils of this study were not fresh and could not be stored for a long period of time without getting rancid. This is of no nutritional interest to humans, suggesting that they could not be utilized as edible oils which allows it not to be consumed as virgin edible oil. These antioxidants act as scavenger for damaging oxygen free radicals. This therefore indicates the ability of such oils to resist lipolytic hydrolysis and oxidative deterioration.

### 4.5 Calorific value

The calorific values ranged from 265.0 to 280.0 kcal. Generally, the *T. indica* seeds have lower calorific values than other dehydrated fruit pulps of common tree species. The energy value of a food indicates how much energy the human body can gain through metabolism. Sources of food energy are categorized into water, proteins, fats, and carbohydrates which represent virtually all the weight of food, while vitamins and minerals make up a small percentage of the weight. Fats have a higher calorific value than all these other categories mentioned. The calorific values in this study (Table 2) are lower than those documented by Sadiq et al. (2016) and El‐Siddig et al. (2006) and higher than values documented for other species such as *Allanblackia floribunda* (Wilfred et al., 2010) and Baobab dried fruit pulp (PhytoTrade, 2009).

The differences in calorific values of *T. indica* to those documented in the literature are likely to be due to the systems used to measure the categories of foods, differences in plant genetic cultivar, growing conditions, stage of maturity at time of harvest, harvest and postharvesting handling, and storage. According to WHO/FAO (2002), the recommended mean energy intake for both male and female population of age group 18–30 years is 2,800 kcal/day but lower in males and females of 60 years and above. Our study found out that the plant species can contribute to the calorific requirement of the body and its consumption will contribute to the calorific requirements of the body. In order to ensure a calorie‐controlled diet, regular consumption of *T. indica* is recommended.

## 5 CONCLUSION

There were significant differences in the physicochemical composition variables between agro‐ecological zones and land use types. The land use types showed strong correlations with physicochemical properties, while agro‐ecological zones did not show correlations. The *T. indica* pulp contains higher vitamin C (ascorbic acid) than the seed samples. The seed samples, however, contain slightly more amounts of β‐carotenoids than pulp samples. In addition, West Nile agro‐ecological zone and wild land use type recorded superior physicochemical contents than other agro‐ecological zones and land use types. The contents of vitamin C in this study show that the species is a valuable, rich, and exceptional source of vitamin C than most known indigenous and conventional fruits and vegetables. Its deficiency diseases in human such as scurvy and pellagra can be prevented through consumption of *T. indica* pulp and seeds. It is also important to consider improving fatty acid composition of dietary *T. indica* oils to enhance health and safety. The documented results thus offer scientific basis for use of *T. indica* pulp and seeds, especially the latter which are always discarded but are nutritionally important for human and animal diets. With a deliberate nutrition education efforts on the benefits of the species, the wild population that showed superior qualities than on‐farm species requires more protection from anthropogenic activities that lead to the loss of these population.

## CONFLICT OF INTEREST

The authors declare no conflict of interests.
